# Upper Extremity Pseudoparalysis: A Case Report of Bilateral Nursemaid’s Elbows

**DOI:** 10.7759/cureus.71801

**Published:** 2024-10-18

**Authors:** Alexander R Orta, Guo Qian, Andrew S Outlaw

**Affiliations:** 1 Emergency Medicine, Alabama College of Osteopathic Medicine, Dothan, USA; 2 Internal Medicine, Alabama College of Osteopathic Medicine, Dothan, USA; 3 Emergency Medicine, Southeast Health Medical Center, Dothan, USA

**Keywords:** atypical presentation, bilateral radial head subluxation, central cord syndrome, nursemaid's elbow, pediatric emergency medicine, pediatric musculoskeletal injury, pseudoparalysis, radial head subluxation, sciwora

## Abstract

Radial head subluxation (RHS), commonly known as 'nursemaid’s elbow,' typically presents in pediatric patients with acute-onset unilateral arm pain and pseudoparalysis. The classic mechanism of injury involves a longitudinal traction force pulling the radial head through the annular ligament. Bilateral RHS is exceedingly rare, with only a few cases documented in the literature; notably, all cases with a provided history and physical exam presented with the classic mechanism of injury, aiding in diagnosis and treatment. Here, we report the case of a two-year-old male with bilateral RHS who presented to the emergency department with bilateral upper extremity pain and weakness following an unwitnessed injury. This case required a broad differential diagnosis, including musculoskeletal injury, central cord syndrome, and spinal cord injury without radiographic abnormality (SCIWORA). It underscores the importance of considering RHS in differential diagnoses, even in atypical presentations, to ensure prompt and effective management.

## Introduction

"Nursemaid's elbow," or radial head subluxation (RHS), is a musculoskeletal injury defined as the subluxation of the radial head through the annular ligament. RHS most frequently occurs in pediatric patients aged 1 to 3 years, with over 20,000 cases reported annually in United States emergency departments [1]. RHS is more common in children above the 75th percentile for weight, with a higher incidence in females and a predilection for the left arm [2]. The most common cause is spontaneous or self-induced injury, with secondary causes involving parents, guardians, and siblings [1]. Bilateral RHS is exceedingly rare, with only a few cases documented in the literature; notably, all cases with a provided history and physical exam presented with the classic mechanism of injury, aiding in diagnosis and treatment [3,4]. This report details a case of bilateral RHS in a two-year-old male in the setting of an unwitnessed injury, illustrating the complexities involved in diagnosing this condition when typical injury mechanisms are not observed.

## Case presentation

**Figure 1:**
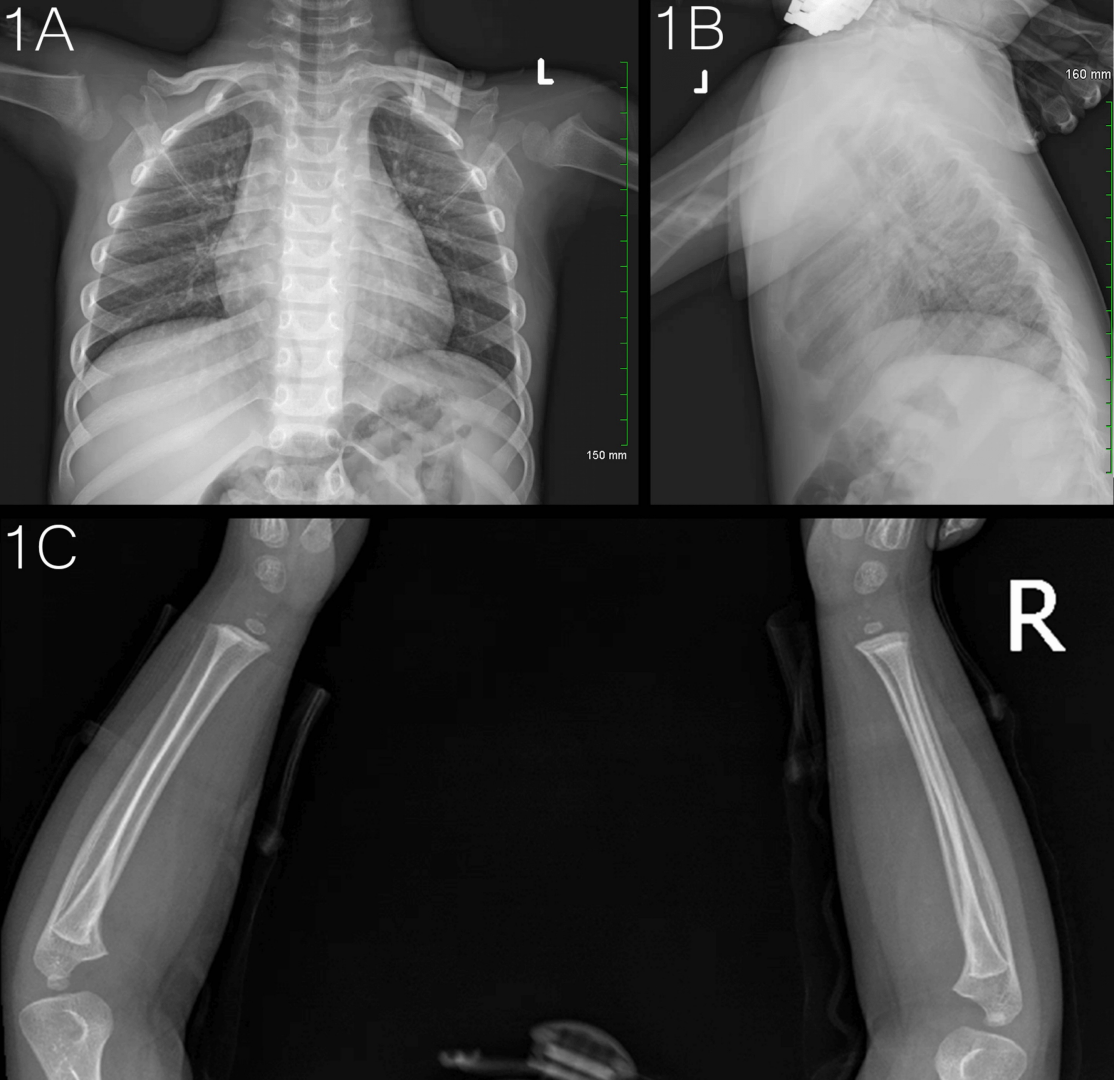
Chest and forearm plain radiographs. Chest AP view (1A, top left); chest lateral view (1B, top right); forearms lateral view (1C, bottom center).

**Figure 2:**
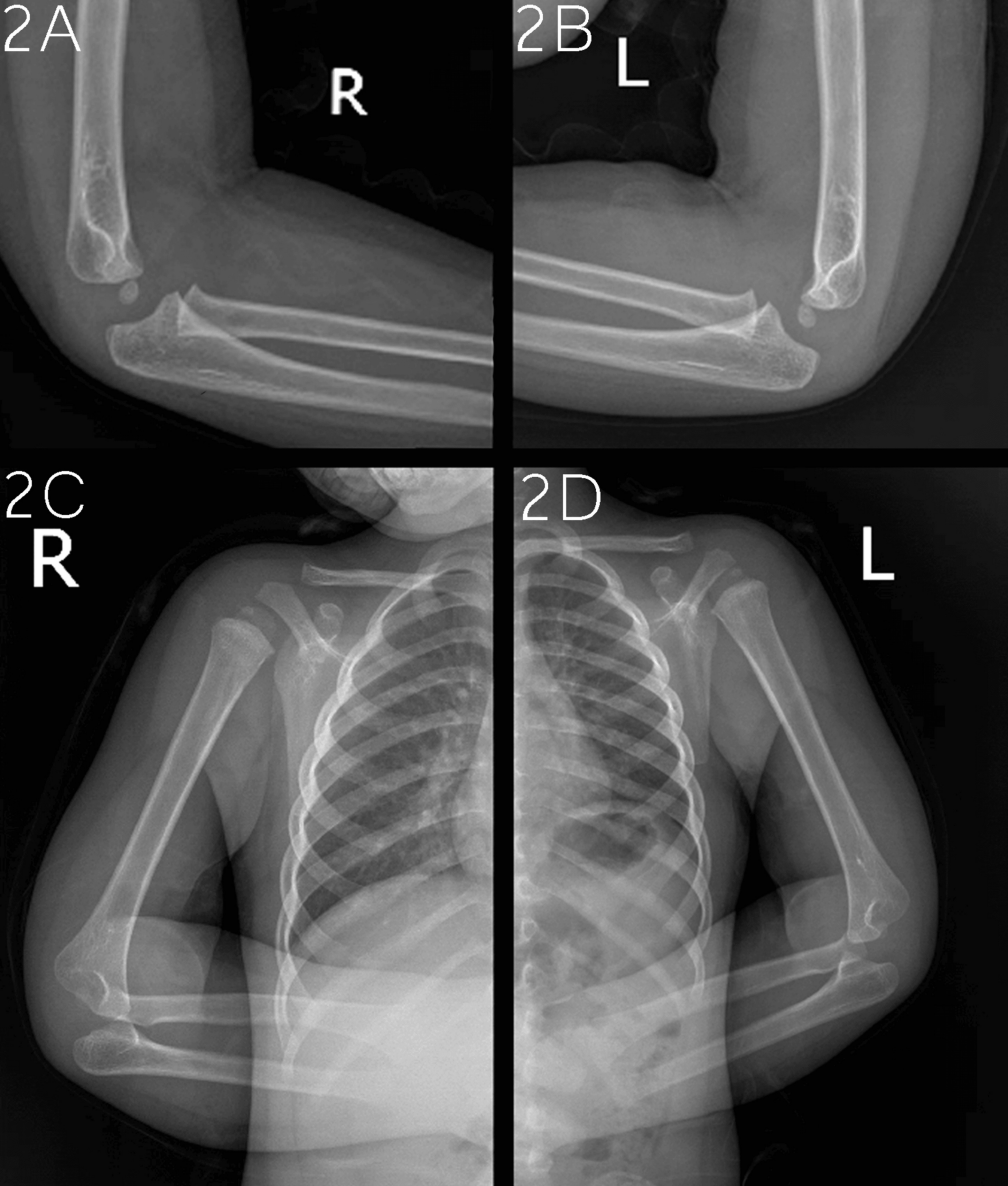
Elbows and humeri plain radiographs. Right elbow lateral view (2A, top left); Left elbow lateral view (2B, top right); Right humerus lateral view (2C, bottom left); Left humerus lateral view (2D, bottom right).

A 2-year-old male presented to the ED following an unwitnessed injury. The child had been dropped off at daycare earlier in the morning in his usual state of health. Later that morning, the caretaker briefly left the room, returning to find the child crying with flaccid upper extremities. Upon picking up the child, the parent observed an absence of response to painful stimuli in the upper extremities, prompting an urgent evaluation at an outpatient clinic specializing in bone and joint injuries. Extensive plain radiographs, including views of the cervical spine, humeri, elbows, and forearms, revealed no evidence of acute fracture or dislocation (Figures 1-2). Due to concerns about neurological injury, a pediatric consultation was sought, and the patient was referred to the ED for further evaluation.

On initial examination, the patient’s vital signs were as follows: heart rate of 117 beats per minute, blood pressure of 102/66 mmHg, temperature of 98.6°F, respiratory rate of 26 breaths per minute, and an oxygen saturation of 97% on room air. The child was alert, well-developed, and in no acute distress. Musculoskeletal examination demonstrated discomfort with passive abduction at the shoulders without bony crepitus or joint deformities. Neurological examination revealed intact sensation to painful stimuli and 0/5 strength in the bilateral upper extremities, with the exception of some finger movement when objects were already in hand. There was no bony tenderness in the bilateral upper extremities or along the cervical, thoracic, or lumbar spine. Skin inspection revealed no ecchymosis, abrasions, or swelling. The chest wall was non-tender, and the head was normocephalic and atraumatic.

Given the absence of overt injuries and concern for neurological involvement, further evaluation included a CT of the head and cervical spine without contrast and a plain-film chest radiograph, all of which were negative for acute findings. During reassessment, an attempt to supinate and flex the right elbow resulted in a palpable click and rapid resolution of symptoms. The same reduction maneuver was then performed on the left arm, again producing a palpable click and resolution of symptoms. With parental consent, no further workup or management was pursued, and the patient was discharged in stable condition.

## Discussion

The classic mechanism of RHS involves a sudden pull on a toddler’s hand or wrist, leading to an abrupt onset of pain and refusal to use the arm (pseudoparalysis) [5]. However, as previously mentioned, the most common cause is spontaneous or self-induced injury, with secondary causes involving parents, guardians, and siblings [1]. One study in cadaveric models demonstrated that a mere 28.5 Newtons of force is required to subluxate the radial head, supporting the proposed injury mechanisms [6].

Clinically, children present with the affected arm held close to the body, the elbow slightly flexed, and the forearm pronated. Diagnosis is primarily clinical, with plain radiographs reserved for cases where a fracture is suspected. Treatment typically involves reduction through supination and flexion at the elbow or hyperpronation of the forearm, often resulting in a palpable or audible click. Following successful reduction, patients generally return to normal use of the arm within 5-10 minutes. Recurrent subluxation is not uncommon, with recurrence rates of approximately 20% [5].

This case of bilateral RHS in a two-year-old male highlights the diagnostic complexities of pediatric upper extremity weakness, particularly when the mechanism of injury is unknown. The presentation necessitated a broad differential diagnosis that included serious conditions such as proximal humerus fractures, central cord syndrome, and spinal cord injury without radiographic abnormality (SCIWORA). With acute fractures excluded on imaging, central cord syndrome and SCIWORA remained significant considerations.

Central cord syndrome

Central cord syndrome is characterized by greater motor impairment in the upper extremities than in the lower extremities due to the anatomical arrangement of corticospinal tract fibers within the spinal cord. This condition typically results from hyperextension injuries in older patients with cervical spondylosis, but it can also occur in pediatric patients following trauma [7,8]. Clinically, it presents with bilateral arm weakness, potential sensory deficits, and variable bladder dysfunction [7]. In this case, bilateral upper extremity weakness raised suspicion for central cord syndrome, but the absence of lower extremity involvement or broader sensory deficits made this diagnosis less likely. Additionally, CT imaging of the cervical spine showed no evidence of spinal cord compression or other typical features of central cord syndrome, although MRI would be required for definitive evaluation [9].

SCIWORA

SCIWORA is typically considered in pediatric patients presenting with neurological deficits post-trauma without evidence of fracture or dislocation on plain radiographs or CT scans. The condition arises from the elasticity of a child’s spine, which allows for significant movement without bony injury but poses a risk of spinal cord damage [10]. SCIWORA was a significant concern due to the child’s upper extremity weakness and the absence of definitive radiographic abnormalities, aligning with the variable nature of SCIWORA presentations. Although MRI was planned for definitive diagnosis, the rapid resolution of symptoms following reduction maneuvers of the elbows confirmed a more benign etiology.

Clinical lessons and broader implications

This case highlights the importance of maintaining a broad differential diagnosis in pediatric patients presenting with unexplained bilateral upper extremity weakness. The inclusion of central cord syndrome and SCIWORA in the initial differential was crucial given the severity of the potential outcomes associated with these conditions. However, the eventual diagnosis of bilateral RHS, confirmed by simple reduction maneuvers, emphasizes the need for emergency physicians to consider both serious and benign conditions in their evaluations. The case also underscores the value of reassessment and clinical vigilance in the emergency department.

## Conclusions

Bilateral upper extremity weakness can be an atypical manifestation of RHS in pediatric patients, especially when the mechanism of injury is unknown. Identifying and managing bilateral RHS requires a broad differential diagnosis to rule out life-threatening injuries and a high level of clinical suspicion to consider more benign causes based on clinical assessment. This approach ensures that all potential causes of injury are considered, leading to timely and appropriate treatment.
